# Wound care practices across two acute care settings: A comparative study

**DOI:** 10.1111/jocn.15135

**Published:** 2019-12-27

**Authors:** Brigid M. Gillespie, Rachel Walker, Frances Lin, Shelley Roberts, Anne Eskes, Jodie Perry, Sean Birgan, Paul Nieuwenhoven, Elizabeth Garrahy, Rosalind Probert, Wendy Chaboyer

**Affiliations:** ^1^ School of Nursing & Midwifery Health Griffith University Gold Coast QLD Australia; ^2^ Nursing Gold Coast Hospital and Health Service Queensland Health Gold Coast QLD Australia; ^3^ Division of Surgery Nursing Princess Alexandra Hospital Brisbane Qld Australia; ^4^ School of Dietetics & Nutrition Gold Coast Hospital and Health Service Health Griffith University Gold Coast Qld Australia; ^5^ Nursing Amsterdam Medical Centre Amsterdam The Netherlands; ^6^ Integrated & Ambulatory Services Nursing Gold Coast Hospital and Health Service Gold Coast QLD Australia; ^7^ Surgical and Procedural Services Nursing Gold Coast Hospital and Health Service Gold Coast QLD Australia; ^8^ Stomal Therapy and Wound Management Department Nursing Princess Alexandra Hospital Brisbane Qld Australia

**Keywords:** acute care, clinical guidelines, documentation, evidence‐based practice, hospitals, nurses, nursing practice, post‐operative care, surgical nursing, wound care

## Abstract

**Aims and objectives:**

Describe and compare current surgical wound care practices across two hospitals in two health services districts, Australia.

**Background:**

Surgical site infections (SSI) are a complication of surgery and occur in up to 9.5% of surgical procedures, yet they are preventable. Despite the existence of clinical guidelines for SSI prevention, there remains high variation in wound care practice.

**Design:**

Prospective comparative design using structured observations and chart audit.

**Methods:**

A specifically developed audit tool was used to collect data on observed wound care practices, documentation of wound assessment and practice, and patients’ clinical characteristics from patients’ electronic medical records. Structured observations of a consecutive sample of surgical patients receiving wound care with a convenience sample of nurses were undertaken. The manuscript adheres to the Strengthening the Reporting of Observational Studies in Epidemiology (STROBE) Statement.

**Results:**

In total, 154 nurses undertaking acute wound care and 257 surgical patients who received wound care were observed. Across hospitals, hand hygiene adherence after dressing change was lowest (Hospital A: 8/113, 7%; Hospital B: 16/144, 11%; *χ* ^2^: 8.93, *p* = .347). Most wound dressing practices were similar across sites, except hand hygiene prior to dressing change (Hospital A: 107/113, 95%; Hospital B: 131/144, 91%; (χ^2^: 7.736, *p* = .021) and use of clean gloves using nontouch technique (Hospital A: 88/113, 78%; Hospital B: 90/144, 63%; *χ* ^2^: 8.313, *p* = .016). The most commonly documented wound characteristic was wound type (Hospital A: 43/113, 38%; Hospital B: 70/144, 49%). What nurses documented differed significantly across sites (*p* < .05).

**Conclusions:**

Clinical variations in wound care practice are likely influenced by clinical context.

**Relevance to clinical practice:**

Using an evidence‐based approach to surgical wound management will help reduce patients’ risk of wound‐related complications.

## 1 INTRODUCTION

Wound care is delivered in a multidisciplinary team; however, it is predominantly a nurse‐led activity. A surgical wound is defined as “a wound created when an incision is made with a scalpel or other sharp cutting device and then closed in the operating room by suture, staple, adhesive tape, or glue and resulting in close approximation to the skin edges.” (p.10), (World Health Organization [WHO], 2016). Surgical wounds are the most common wounds seen in hospitals, with one surgical procedure yearly for every 22 people worldwide (Lancet Commission on Global Surgery, 2018). Surgical site infections (SSIs) are complications associated with any surgical procedure (Berrios‐Torres et al., 2017), yet they are the most preventable hospital‐acquired infection (Allegranzi et al., 2011). Some experts estimate that up to 9.5% of inpatient surgical procedures will experience a SSI (European Centre for Disease Prevention, 2013; Mangram, Horan, Pearson, Silver, & Jarvis, 1999). SSI represents a significant burden relative to patient morbidity and mortality and additional costs to healthcare systems worldwide. Current estimates indicate that wounds account for almost 4% of total healthcare system costs and that proportion is increasing (Lee, Agarwal, Lee, Fishman, & Umscheid, 2010). Therefore, it is imperative that pre‐ and postoperative management is of high quality, based on the best available evidence.

## 2 BACKGROUND

Over the past 20 years, recommendations in clinical practice guidelines (CPGs) for SSI prevention have been developed and updated to reflect advances in the evidence base (Gillespie et al., 2018). Despite this, there remains inconsistency in the use of CPGs, contributing to great variability (Brölmann et al., 2012; Gillespie, Chaboyer, St John, Morley, & Nieuwenhoven, 2014; Lin et al., 2018) in wound care practices globally. Additionally, variance in the quality of evidence that underpins recommendations coupled with the fact that very few recommendations pertain to wound care strategies (Brölmann et al., 2012; Gillespie et al., 2018) contributes to uncertainty and a lack of standardisation in clinical practice. It is often accepted that wound care practices are influenced by various local factors, and not just the evidence. For instance, the resources available, skill mix, clinician preferences, patient behaviour and length of hospital stay contribute to practice variation (Sutherland & Levesque, 2019). A systematic review of medical practice variation in developed countries identified 836 published studies and detailed variation across regions, hospitals and physician practices in almost every surgical field, condition and procedure (Corallo et al., 2014). While variation in wound care is widely acknowledged from an international perspective (NICE, 2008; WHO, 2016; Wounds Australia, 2017), there is a paucity of research to describe contemporaneous surgical wound care practices, and there are little, if any, comparative data available.

A recent systematic review (Gillespie et al., 2018) critically evaluated the quality of CPGs for SSI prevention using the Appraisal of Guidelines for Research and Evaluation II (AGREE II) tool (Brouwers et al., 2010). Notably, most SSI prevention CPGs scored lowest in the domain of “applicability,” suggesting that implementation of the guideline was challenging across different clinical settings (Gillespie et al., 2018). Notably, the local context has a bearing on how guidelines are integrated into clinical practice, although contextual differences are not well described (Gillespie et al., 2018). Ideally, clinical practice should be based on the best available evidence, combined with patients’ preferences, and accounting for the local context, resources and skills (Brölmann et al., 2012).

The aim of this study was to describe and compare current surgical wound care practice across two hospitals in Queensland, Australia. Gathering evidence on variation in practice provides a foundation to explore where this variation occurs and why. Understanding variation in practice has been a foundation upon which low‐value care is identified and discontinued; ultimately, an aim of the “Choosing Wisely” campaign.

## 3 METHODS

A prospective comparative design using structured observations and electronic health record (EHR) audit. The study is reported according to the Strengthening the Reporting of Observational Studies in Epidemiology (STROBE) Statement (Little et al., 2009; see Appendix S1).

The study setting was two large metropolitan hospitals across two health services districts in Queensland, Australia. Hospital A is a tertiary university hospital with 750 beds, has six surgical units and performs approximately 18,000 surgeries per year. This facility provides specialised care in all surgical specialties except organ transplantation. Hospital B is a quaternary facility with 800 beds, performs 21,000 surgeries annually, has 11 surgical units and provides highly specialised care except for obstetrics and gynaecology.

In all, we invited 17 surgical units where simple and complex wound care was undertaken to participate across both hospital sites. The sampling frame for the observations included ward nurses and postoperative patients. We used consecutive sampling of wound care episodes undertaken by nurses. We had planned a priori to undertake up to 150 observations of wound care at each hospital but the type of information being collected was similar, with redundancy at Hospital A after the first 110 observations, and Hospital B, after 140 observations.

We modified an observational tool previously developed and rigorously tested in a pilot study (Ding, Lin, Marshall, & Gillespie, 2017) to audit contemporaneous wound management practices in the participating hospitals. Items in the audit tool included postoperative surgical wound management behaviours/activities used to prevent infection. The audit tool included demographic and wound characteristic‐related questions to describe the sample of patients (i.e., gender, age, comorbidities, surgical specialty/procedure, length of surgery, wound type/location). Surgical wounds were classified as being either simple or complex. Simple wounds occur suddenly and follow the normal wound‐healing pathway (Berrios‐Torres et al., 2017). Complex wounds are characterised by extensive loss of integument, infection, compromised viability of superficial tissues and/or associated systemic pathologies that impair the normal healing process (Ferreira, Júnior, Carvillo, & Kamamoto, 2006). These questions were answered using information from patients’ EHR. The audit tool also included free text boxes to document clinical information and/or activities pertinent to the episode of wound care observed. Registered nurses undertaking wound care were asked to provide demographic data relating to age, nursing experience, level of education, role and employment status.

Six experienced registered nurses performed all structured observations at the two sites. The nurses did not work on the wards where the observations were undertaken, thus were independent observers. Two of the authors trained the data collectors and undertook some observations with them to establish consistency in recording and interpreting observations. Observers also used a data dictionary, based on several clinical practice guidelines relating to SSI prevention that included hand hygiene, wound cleansing and general asepsis—viz, the NICE (2008), NICE (2013) and WHO (2016) and the CDC (Berrios‐Torres et al., 2017; Mangram et al., 1999) CPGs. Consistency among trainers and trainee observers was assessed by calculating the proportion of agreement, which averaged 76%–90%.

During observations, nurses could be observed more than once however patients were observed only once. Structured observations were undertaken during weekdays, as this is when the bulk of surgical activity occurred. Observational and EHR data were collected over a four‐month period during 2017–18.

Approval for the study was given by the relevant university and hospital Human Research Ethics committees. Nurse participants completed a brief demographic profile while patients gave permission to access their EHR. All study participants signed a consent form.

Data were entered into SPSS (v24; IBM) and checked for accuracy using a random sample of 20%. Descriptive statistics were used to calculate absolute (*n*) and relative frequencies (%), means and standard deviations (*SD*) or medians and interquartile range (reported as upper and lower quartiles), as appropriate to the level of data and its distribution. Inferential comparisons were computed using the Chi‐squared (*χ* ^2^) statistic, Mann–Whitney *U* test or independent samples *t* tests, as determined by the level of the data and its distribution. Statistical significance was set at *p* < .05.

## 4 RESULTS

Across the two sites, 154 nurses were observed while undertaking acute wound care. Most nurse participants in the combined sample were female (130/154, 84.4%). The median age across the sample was 28.0 years (IQR 12.0, range 21–58 years). Median years of clinical experience in nurses’ current clinical role were 3.9 years (IQR 5.0, ranged from 1–25 years). Almost three quarters of nurses in the combined sample reported having a Bachelor's degree as their highest qualification (112/154, 73%). Across the sample, 8/154 (5.2%) nurses worked in advanced practice roles as a Clinical Nurse Consultant. Table 1 details nurse participants’ demographic characteristics for each site relative to gender, age, qualifications, clinical role and employment status. Across hospitals, there were no significant differences except for gender (*p* = .044).

**Table 1:** Nurse demographic characteristics (*n* = 154)

Demographic characteristic	Hospital A	*n* = 56	Hospital B	*n* = 98	*p*‐Value
	*n*	%	*n*	%	
Gender^a^					
Female	51	91.1	79	80.6	.044^b^
Male	4	7.1	19	19.4	
Highest qualification^a^					
Undergraduate	45	81.8	87	89.7	.120^b^
Postgraduate	10	18.2	10	10.3	
Employment status^a^					
Full‐time	14	25.0	30	30.6	.499^b^
Part‐time	41	73.2	68	69.4	
Role^a^					
Enrolled nurse	9	16.1	6	5.1	.113^b^
Registered nurse	36	64.2	73	74.5	
Clinical nurse/clinical nurse consultant	11	19.6	19	19.4	

	Median	Interquartile range	Median	Interquartile range	*p*‐Value^c^
Age^a^ (years)	30.0	22–56	28.0	21–58	.122
Clinical experience in current role^a^ (years)	4.0	1–21	3.0	1–25	.113

a <1% missing data.

b *χ* ^2^ test used.

c Mann–Whitney *U* test used.

In total, 257 surgical patients who received wound care by ward nurses were included in the sample. Nearly, two thirds (*n* = 162/257, 63.0%) of patient participants across both sites were male, while the average age of patients was 59.4 years (*SD* = 16.3 years, range 16–91 years). Over half (*n* = 146/257, 58.9%) of patients in the combined sample had undergone elective surgery while 71/257 (27.6%) and 31/257 (12.5%) of patients had emergency (patient's condition requiring surgery within 10 days) and emergent (patient's condition requiring surgery within 24 hr) surgeries, respectively. Across the entire sample, 90/257 (35.0%) patients were seen by a dietitian and 71/257 (27.6%) received postoperative wound care education. Table 2 shows the breakdown of clinical characteristics of patients for each hospital. Group differences (*p* < .05) across hospital sites were evident in the number of postoperative days, surgical specialty and wound type.

**Table 2:** Patients’ clinical characteristics (*n* = 257)

Demographic characteristic	Hospital A	*n* = 113	Hospital B	*n* = 144	*p*‐Value^a^
	Mean	*SD*	Mean	*SD*	
Age (years)	61.6	16.2	57.8	16.3	.052

	Median	Interquartile range	Median	Interquartile range	*p*‐Value^b^
Length of surgery (minutes)	100.0	4–960	112.5	13–860	.707
Number of postoperative days	7.0	1–28	4.0	1–62	**.0001**

	*n*	%	*n*	%	*p*‐Value^c^
Gender					
Female	38	33.6	57	39.6	.326
Male	75	66.4	87	60.4	
Surgical specialty					
General	33	29.2	19	13.2	**.0001**
Orthopaedic	6	5.3	29	20.1	
Vascular	25	22.1	36	25.0	
Plastics	46	40.7	19	13.2	
Head and Neck surgeries≈	61	54.0	61	42.4	
“Other”	7	6.2	36	25.0	
Surgery classification					
Elective	61	54.0	85	63.0	.152
Emergent	26	23.0	5	3.7	
Emergency	26	23.0	45	33.3	
Wound type					
Simple	74	65.5	111	77.1	**.040**
Complex	39	34.5	33	22.9	
Wound classification					
Clean or clean‐contaminated	102	90.2	134	93.0	.308
Contaminated or dirty or infected	11	9.8	9	7.0	
Comorbidities^d^					
Cardio‐pulmonary diseases	75	63.0	138	95.8	.487
Diabetes	31	27.4	42	29.2	
Cancer	38	33.6	24	16.7	

Note

Bolded *p*‐values <. 05.

≈*Head and Neck surgeries* included neuro, maxillary facial, ear–nose–throat, and eye surgeries; “Other” types pf surgery included gastro‐intestinal, cardiothoracic, trauma, urology, head and neck, breast, transplant, endocrine, hepato‐biliary procedures.

a Independent samples *t* test used.

b Mann–Whitney test used.

c *χ* ^2^ test used.

d More than one comorbidity documented in patients’ EHR; *Cardio‐pulmonary diseases* included ischaemic heart disease; peripheral vascular disease, renal disease, hypertension and chronic‐obstructive airways disease.

**Figure 1:**
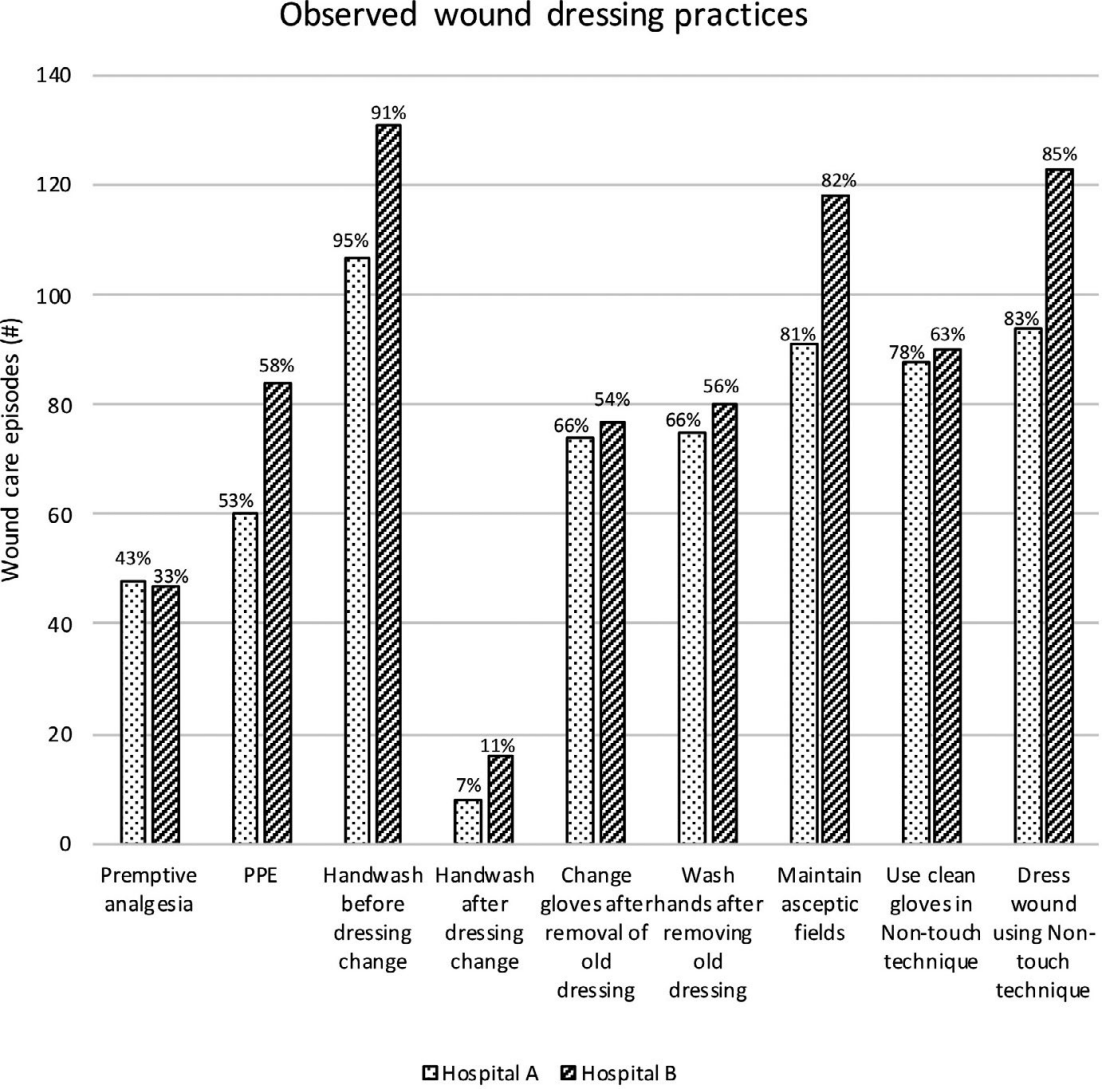
Observed wound dressing practices across Hospitals A (*n* = 113) and B (*n* = 144)

Figure 1 shows comparative results relating to observed wound dressing practices across the two hospitals. Adherence to hand hygiene guidelines was highest prior to dressing change (Hospital A: 107/113, 95%; Hospital B: 131/144, 91%), but was lowest after dressing change (Hospital A: 8/113, 7%; Hospital B: 16/144, 11%). There were statistically significant differences between hospitals in relation to hand hygiene adherence (*p* = .021), and the use of clean gloves in a nontouch technique (Hospital A 88/113, 78%; Hospital B 90/144, 63%; *p* = .016). There were no statistically significant differences between hospitals in seven of the nine observed wound dressing practices (*p* > .05)

**Figure 2:**
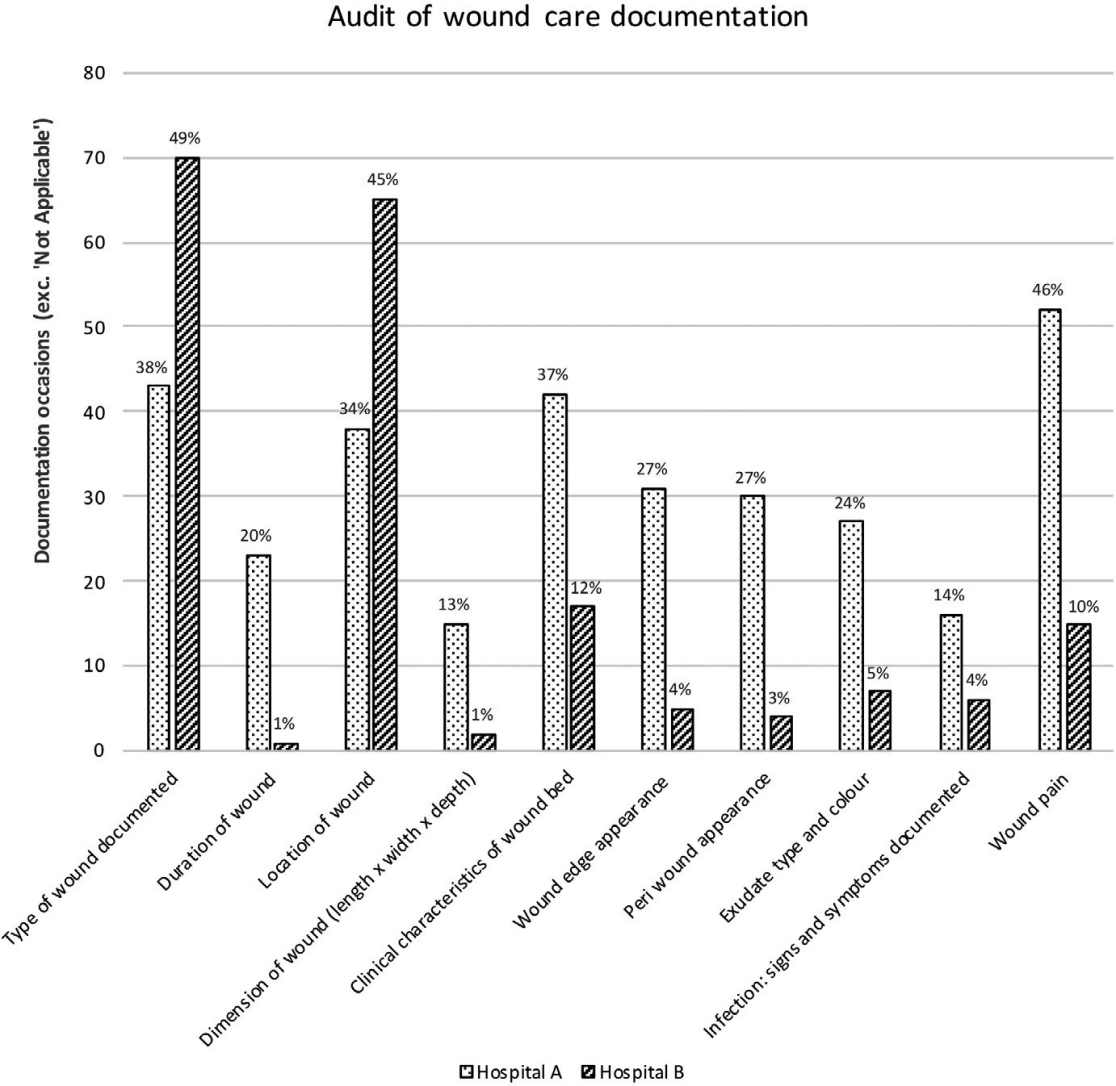
Audit of documentation of wound assessment across Hospital sites

Patient care records were audited for all 257 occasions of observed wound care. Document sources included EHR, bedside chart or wound assessment pathway form, because wound care assessments were documented in one or more places. However, most wound assessments were documented in patients’ EHR progress notes (221/257, 86.0%). Figure 2 shows comparative data in relation to documentation of wound care regime. Across both sites, the most documented wound characteristic was *type of wound* (Hospital A: 43/113, 38%; Hospital B: 70/144, 49%). *Wound location* was the second most common characteristic documented (Hospital A: 38/113, 34%; Hospital B: 65/144, 45%). The differences between sites were statistically significant (*p* < .05) across all 10 aspects of wound documentation (Figure 2).

## 5 DISCUSSION

This study described and compared surgical wound care practices across two tertiary hospitals in Queensland, Australia. Hospital A is co‐located with a major Queensland university while Hospital B has Magnet designated facility, which recognises nursing excellence and high‐quality patient care. Hence, these hospitals are comparable in their philosophies of providing quality patient‐centred care and recognising nursing excellence in clinical practice and education. Nurses observed across sites were similar in relation to important demographic variables (Table 1 **)**; for example, they had similar levels of clinical expertise, education and experience. Across the entire sample, 15% of nurses held a postgraduate qualification and in nursing roles were similar, with only 5% of nurses across sites working autonomously in advanced practice roles. Patient cohorts were similar across hospitals relative to gender, age, length of surgical procedure and across 4/9 surgical specialties (Table 2). However, there were notable differences in patient cohorts in terms of surgery classification and wound type. Generally, patients at Hospital B were more acute, had a higher number of complex surgeries, and had surgical wounds classified as predominantly “simple.”

Overall, there was reasonable consistency in nurses’ wound dressing practices, except for hand washing before dressing change and using clean gloves in a nontouch technique (Figure 2). The reasons for these anomalies across sites are unclear, although there were statistically significant differences in surgical case mix relative to specialty (Table 2) and procedure across four specialties. In this study, the low hand hygiene adherence rates (9.3% overall) following dressing change is a major departure from infection prevention recommendations, which is somewhat concerning. Findings from this study support the results of earlier research, suggesting incongruences in hand hygiene practices *before* and *after* patient care (Ding et al., 2017). Yet, in comparing practice variations across contexts, it is important to separate contextual factors that are outside the direct control at that level and those that are amenable to change (Sutherland & Levesque, 2019). Clearly, inconsistencies in practices may be due to environmental factors outside of the control of healthcare providers, viz, time constraints, skill mix, workload and patient acuity. A recent qualitative study reported distractions from other staff and patients can reduce hand hygiene adherence after wound care (Lin et al., 2018). Hand hygiene is critical in the application of standard precautions (Berrios‐Torres et al., 2017) and reduces the patient's risk of developing a SSI (Lin et al., 2018).

Our results showed site differences in nurses’ use of clean gloves in a nontouch technique during dressing procedures to prevent wound contamination. Results of earlier research on postoperative wound dressing practices found that strategies used to reduce wound contamination were not always evidence‐based, and nurses were often unable to provide a rationale for their actions (Bree‐Williams & Waterman, 1996). Arguably, the use of “aseptic technique” in postoperative wound care is historically based on ritualistic and entrenched practices in the absence of an evidence base and has often been carried out without question (Bree‐Williams & Waterman, 1996; Gillespie & Fenwick, 2009). Results of an earlier systematic review indicate there is limited scientific research to support many wound care practices currently undertaken in acute care environments (Brölmann et al., 2012). As such, nurses may be undertaking time‐consuming practices that do not necessarily benefit surgical patients. Despite the substantial body of evidence in wound care, high‐level evidence to guide practice and treatment decisions remains scarce (i.e., randomised controlled trials and meta‐analyses; Brölmann et al., 2012; Ubbink, Brolmann, Go, & Vermeulen, 2015). Indeed, the absence of high‐quality evidence in this area means that clinicians rely on alternative sources of information to inform the wound care they give (Gillespie, Chaboyer, St John, et al., 2014). Frequently, treatment decisions in surgical wound care are based on personal opinion, experience, the preferences of health professionals and the biases of wound product manufacturers (Brölmann et al., 2012; Gillespie, Chaboyer, Niewenhoven, Chaboyer, Niewenhoven, & Rickard, 2012; Gillespie, Chaboyer, St John, et al., 2014). Some experts assert a combination of individual, interprofessional and contextual factors, such as hospital/department culture perpetuate the use of low‐value care (Harvey & McInnes, 2015). Plausibly, the role of cognitive biases such as “therapeutic allusion” may result in the overuse of ineffective care or the underuse of effective care (Cott, Soon, Elshaug, & Lindner, 2017).

Our results show significant differences in all aspects of documented wound care (Figure 2) across hospitals. These differences may be due partly to contextual variances relative to how clinical data are collected in the documentation systems used in each hospital. For example, Hospital A’s documentation system, while available electronically, at the time of this study used a traditional paper‐based method, where the data were scanned into an electronic database. Conversely, Hospital B used an interactive documentation system that is completely integrated. To be effective, the integrated EHR relies on data being entered into specific sections (e.g., Power Forms), yet the interface is often sparsely populated because of the time it takes nurses to complete each section electronically. A recent systematic review (Baumann, Baker, & Elshaug, 2018), on the introduction of fully integrated EHR found statistically significant differences in the proportion of staff time spent on clinical documentation; the proportion of time spent was higher in settings where EHR systems were used compared to hospitals without EHR (Baumann et al., 2018). Clearly, healthcare professionals generally choose more traditional formats (e.g., progress notes vs. clinical pathways) to document clinical information. Results of the current study suggest differences exist between sites relative to the *content* nurses documented. Our findings are consistent with a recent study that reported nurses often used their own judgement on where and what to report in relation to patients’ wounds (Lin et al., 2018). Differences across hospitals in this study may also be due to environmental factors. Findings from a recent qualitative study describing the barriers and enablers to wound documentation suggest that time constraints and clinical workloads contribute to the lack of completeness in EHR (Lin et al., 2018).

Differences in patient populations may also account for the lack of documentation noted in Hospital B. For instance, a higher proportion of patients in this facility had simple wounds (77%) and underwent orthopaedic procedures (20%), where standard practice is to leave the dressing intact for up to five days as per their clinical pathway. As such, this patient cohort would have had minimal wound care during the postoperative period, so the descriptors used to signify documentation of various aspects of wound care are, in some cases, not applicable. Saliently, the absence of wound care (e.g., because of the need for the dressing/bandage to remain intact) and the reasons for this are important to communicate. The results of an earlier chart audit study indicated that only 40%–75% of postoperative wound care episodes were documented (Gillespie, Chaboyer, Kang, et al., 2014). While the incidence of SSI is the predominant indicator used to measure the quality of surgical care (NICE, 2008), wound care documentation is also an indicator, upon which care can be benchmarked against other similar facilities. Notably, documentation of practice mitigates clinical and legal risk (Staunton & Chiarella, 2013) and has patient safety implications for communicating continuity of care (Gillespie, Chaboyer, Kang, et al., 2014). Yet, anecdotally we know many other health professions do not read nurses’ notes. Thus, while we note differences across sites, understanding the extent to which variations in wound care documentation is a patient safety risk is an area to be explored.

We acknowledge several limitations to this study. First, although two hospital sites were included, using volunteer wards may have resulted in selection bias and limited generalisability. Second, while it is one of the few prospective studies in its field, it is possible ward nurses may have changed their practice in response to being observed (i.e., Hawthorne effect). Further, the accuracy and completeness of the documentation audited may not indicate the extent to which practices were actually undertaken. However, triangulating these two methods of data collection yielded a more comprehensive understanding of contemporaneous wound care practice across the two sites. Finally, different data collectors performed structured observations at each hospital site and there was modest variability in consistency among trainees and trainers during training for observations. To minimise the variation in interpretation of practices observed, we developed a data dictionary and the observers were experienced registered nurses.

## 6 CONCLUSION

The results of this study have raised pertinent questions relative to contextual factors and potential cognitive biases that enable or inhibit nurses’ ability and willingness to use an evidence‐based and standardised approach to wound management and documentation. Given recommendations from CPGs are based on variable quality of evidence, it seems important for future work to better understand which practices are potentially low value. Until we have higher quality evidence for some surgical wound practices, it is likely variation will occur and we will not know how much of this variation reflects potentially wasteful (i.e., low value) care.

## 7 RELEVANCE TO CLINICAL PRACTICE

Our results suggest some variability in surgical wound care practice across the participating hospitals. Inevitably, there will always be differences across clinical settings, depending on the organisational context, the people involved and the types of patient cohorts managed in these facilities. Postoperative wound care has a significant impact on pain, suffering and minimises the effect of wound complications such as infection. Using an evidence‐based framework and a standardised approach may reduce the risk of wound chronicity.

## CONFLICT OF INTEREST

The authors declare that they have no conflicts of interest.
